# Sheltering from the storm: The role of a quality improvement collaborative focused on improving HIV care retention in New Orleans during and following Hurricane Ida

**DOI:** 10.1371/journal.pone.0323026

**Published:** 2025-05-09

**Authors:** Emily A. Arnold, Shannon Fuller, Bruce D. Agins, Lauren Fidelak, Jessica Xavier, Wayne T. Steward, Greg M. Rebchook, Kimberly A. Koester

**Affiliations:** 1 Department of Medicine, University of California San Francisco, San Francisco, California, United States of America; 2 Department of Health, Behavior and Society, Johns Hopkins Bloomberg School of Public Health, Baltimore, Maryland, United States of America; 3 Department of Epidemiology and Biostatistics, University of California San Francisco, San Francisco, California, United States of America; 4 Gillings School of Global Public Health, University of North Carolina, Chapel Hill, North Carolina, United States of America; UT Southwestern: The University of Texas Southwestern Medical Center, UNITED STATES OF AMERICA

## Abstract

**Background:**

Climate-related events, including hurricanes in New Orleans, Louisiana (NOLA), are becoming more intense and frequent, presenting challenges to HIV-related health care system facilities, staff, and patients. Addressing these challenges through quality improvement collaboratives (QICs) may blunt their impact through redesign of systems, leading to improved retention in HIV care during severe disruptions. We present a case study of a federally-supported QIC that was in progress to improve care engagement when Hurricane Ida struck in August 2021.

**Methods:**

We conducted key informant interviews with providers, health department staff, and capacity building specialists involved in the QIC. These interviews were augmented by fieldnotes from 4 learning sessions, day-long workshops that featured clinic-level and jurisdiction-level presentations and brainstorming, including efforts to improve emergency preparedness and respond to hurricanes.

**Results:**

Immediate disruptions included damage and loss of physical buildings, homes, electrical power, and displacement of clients and providers/staff. These disruptions contributed to significant barriers to accessing HIV care and treatment. Efforts to overcome barriers resulted in solutions to help clients gain access to HIV treatment, especially at pharmacies in nearby states, to which they were evacuated or voluntarily relocated. Being part of the QIC and using QIC-related listservs and messaging apps allowed HIV-care teams to more effectively connect with clients, facilitate access to treatment, and feel less isolated as they navigated the hurricane’s impact. Although the QIC was not originally focused on climate change or disaster relief, following the hurricane, the necessity of addressing emergency preparedness became clear to ensure continuity of care during climate-related events.

**Conclusions:**

There is an urgent need to build health care system resiliency against climate-related disasters and emergencies. QICs offer a vehicle for strengthening systems and assuring quality of care to address disasters and prepare for them, crises that are unfortunately ever more frequent as climate change advances.

## Introduction

In the United States, the HIV epidemic is concentrated in the Southeast geographical region, where there are fewer HIV-specific resources, low-income patients are less likely to have public forms of health insurance and those impacted are most likely to be living in poverty and minoritized [1–3]. New Orleans, Louisiana (NOLA) is one of the Ending the HIV Epidemic jurisdictions, with approximately 7,000 people living with HIV residing in the jurisdiction [4]. Approximately 65% of people living with HIV in NOLA are virally suppressed, which is well below the 90-90-90 target [4]. A key part of reaching viral suppression is having individuals retained in care. As part of a federally-funded initiative [5], the NOLA jurisdiction launched a 4-year quality improvement collaborative (QIC) to improve HIV care retention in 2019.

Climate-related events are becoming more intense and frequent, presenting challenges to the HIV-related health care system in terms of their impacts on facilities, staff, as well as on patients themselves [6–10]. These events have been intensifying in the Southeast over the past decade, particularly hurricanes, flooding, and extreme heat waves [11]. Although research on the association between climate-related events and HIV care-related health outcomes is limited, a recent study from Miami [12] showed that people living with HIV (PLWH) were likely to not attend clinic appointments during days with extreme weather events. Research from California has also documented the impact of climate-related events, such as wildfires, on the displacement of PLWH as well as clinic staff and providers [6,7].

Hurricanes have long been seasonal events in NOLA and across the Southern US, but their frequency and intensity has increased over the past decades [13]. Since Hurricane Katrina in 2004, the city of New Orleans has invested significant time and resources in emergency planning and infrastructure to more effectively prepare for and respond to these natural disasters. New Orleans itself lies below sea level and is surrounded by water, making it especially vulnerable to intense storms, flooding, and sea level rise. Hurricane Ida, which made landfall in Louisiana on August 29^th^, 2021, is the sixth costliest storm in US history, with $75B in damages, $18B in Louisiana alone [14]. Ida hit New Orleans on the anniversary of Hurricane Katrina, rapidly increasing from a Category 1 storm to a Category 4 storm, with 150 mph winds [15]. Given how quickly the storm intensified, Mayor LaToya Cantrell issued a mandatory evacuation order for all parts of the city outside of flood protection areas somewhat late, on August 28^th^. Hurricane Ida brought a storm surge and rain, power outages and 36 tornadoes across the Atlantic coast [16]. In Louisiana, 30 people died during the storm. In New Orleans, the entire city lost power when its major power supplier, the 400 foot-tall Entergy tower, collapsed [17]. Schools lost roofs, the Mississippi river reversed course following the storm surge, and ferries ran aground. Levees were also overcome in some places causing residents to flee floodwaters.

Sweltering heat, lack of food, disrupted communication channels, and broken supply chains exacerbated the direct impact of the storm in its immediate aftermath. Due to the late issuance of an evacuation notice, many people with low incomes could not leave during Ida because of insufficient resources, including lack of personal transportation and money; approximately 200,000 residents stayed [15,18]. Power was not restored for at least ten days, longer in some places. Without power for air conditioning, 10 people died due to heat. In addition, more than 250,000 school children were unable to go back to school, garbage service was delayed, and traffic lights were out, some of them for months following the storm. A curfew from 8 pm to 6 am was put in place through September 8^th^. Eventually, the National Guard was deployed to the city to help with recovery efforts along with 25,000 other workers.

Patient retention in care during extreme weather-related events is of particular concern for PLWH. Given how poverty and marginalization affect so many HIV patients, providers struggle to maintain continuous quality care. Moreover, the threat to community health resulting from disruptions in continued access to ART is of great concern given the role of viral load suppression in preventing transmission of HIV. When Ida struck, New Orleans as a jurisdiction was in the process of implementing interventions to assist with retention and re-engagement in care efforts, primarily involving community health workers (CHWs) to find and engage clients in care. This effort was funded by a federal initiative (2019–2023) – the HRSA Special Projects of National Significance “Capacity Building in the Ryan White HIV/AIDS Program (RWHAP) to Support Innovative Program Model Replication Initiative” [5] -- to enhance the capacity of US jurisdictions facing HIV-related disparities to implement evidence-informed interventions to improve HIV care outcomes using a QIC approach [19]. Although originally conceived before the introduction of the national Ending the Epidemic (EHE) Initiative, jurisdictions in this HRSA initiative ended up representing a sample of EHE priority regions based on their high HIV incidence and ongoing disparities in key populations [20]. In partnership with the University of California San Francisco (UCSF), jurisdictions used the QIC model to choose and implement evidence-based interventions. In NOLA, QIC activities focused on increasing patient retention through integrating community health workers in HIV care settings. However, in the wake of Hurricane Ida, the QIC pivoted to improving emergency preparedness, which is the focus of this case study. We present findings from an evaluation of a QIC that was in place when Hurricane Ida hit New Orleans on August 29th, 2021, and explore the role that the QIC had in responding to the immediate and long-term consequences of this disaster, particularly for those living with HIV.

## Materials and methods

### Sample

We conducted qualitative interviews across all participating jurisdictions as part of our multi-method evaluation study. For this paper, we draw from 9 interviews and observations from 4 learning sessions conducted in New Orleans, following Hurricane Ida. We used purposive sampling to recruit and interview key informants who included health department leadership and staff, other QIC Planning Body members, and staff and leaders from participating clinics/agencies. Interviews in NOLA were conducted between September 2021 and February 2022, approximately 1–5 months after Hurricane Ida struck, and 1.5 years since programs had been initiated to integrate CHWs into HIV care engagement efforts. Interview data were augmented by fieldnotes taken at 4 learning sessions, from January 2022-June 2023, which were focused on improving emergency preparedness and responding to Hurricane Ida.

### Data collection

Our study procedures were reviewed by the UCSF institutional review board and received a “not engaged in human subjects research” determination because our work was focused on quality improvement and data were based on perspectives from our participants’ professional capacities. Nonetheless, we followed standard research procedures in recruitment, interviewing, and data protection. Interview participants were recruited via email by a member of the qualitative evaluation team (EA), a female, trained anthropologist and professor with over 20 years of working in the HIV-related field. All of the participants who were approached agreed to be interviewed and received written information about the study. All participants provided verbal informed consent to participate, which was recorded at the outset of the interview, and were offered a $75 gift card to participate. Interviews took place via Zoom and lasted approximately 60–75 minutes.

Semi-structured interview guides were developed by a team of experienced qualitative researchers (EA, SF, KK) and were designed to capture internal and external drivers of implementation and program outcomes (such as appropriateness, acceptability, adoption, feasibility, and sustainability) [21] and guided our larger project’s overall evaluation design. We also explored the perceived impact of the interventions and the QIC. Hurricane Ida, and its overlap with a surge in COVID, represented a pivotal episode for clinics attempting to maintain connections to their clients. As a result, interview conversations included participant narratives about the impact of the hurricane and recovery as a focal point for the QIC, even though that was not an initial focus of the project. The team decided to code and analyze the narratives related to Ida as the storm represented an “external” driver for implementation of the CHW-delivered interventions and their effectiveness in improving retention and engagement in care.

Qualitative researchers (EA, JX, SF, AM) also conducted observations of the QIC learning sessions conducted in New Orleans and recorded field notes. Learning Sessions were one-to-two-day meetings, initially held over Zoom due to the COVID-19 pandemic but later held in person, convening jurisdiction-level leadership, representatives from participating agencies and community members. The qualitative observers and their roles were introduced at each Learning Session. Observations provided a way to gain understanding of the interpersonal relationships and contextual information about the participating sites to assist with data interpretation. Observations also helped to identify potential key informants and follow-up questions for the interviews. For this analysis, the team focused on fieldnotes from 4 Learning Sessions following Ida, which were dedicated to emergency preparedness and climate-related events. The fieldnotes, taken in conjunction with interview transcripts, allowed the team to triangulate our interpretation of the data and achieve a deep understanding of the role of the QIC during and following Ida.

### Analysis

We conducted a thematic analysis [22] of interviews and fieldnotes. First, interviews were audio-recorded and transcribed verbatim. Authors SF, KK, EA, and JX developed a codebook that applied across all the jurisdictions for the larger initiative. The codebook consisted primarily of *a priori* codes based on implementation outcomes and the interview guide, which focused on understanding participant experiences in the capacity-building initiative. We also included codes based on themes that emerged during the data collection process, such as the impact of weather-related events and the COVID-19 pandemic. Authors SF and KK coded all transcripts in Dedoose [23], a cloud-based application that facilitates qualitative data management and extraction of coded excerpts. The research question for this sub-study emerged from the coding process and the lead author’s interest in studying the impact of climate-related events on HIV-related services and the HIV healthcare workforce. For this study, all excerpts under the following codes were reviewed and summarized: weather-related events, the double burden of COVID, QIC narrative, with sub-codes on facilitators and barriers related to intervention implementation. Looking across all the summaries, the lead author prepared an analytic memo that combined information across the key codes. The analysis focused on identifying common themes related to the immediate impact and aftermath of Ida and the role of the QIC in patient care and treatment outcomes. Findings were reviewed and discussed with the qualitative evaluation team, the larger QI team (WTS and BDA) and supplemented with information from the fieldnotes taken during learning sessions.

## Results and discussion

Below, we present the findings from this analysis in chronological order, describing the complexities that HIV-related agencies and clinics faced as Ida hit and the role that the QIC played during and in the aftermath of the hurricane.

### Setting the stage: COVID + Ida double whammy: “One step forward, two steps back”

Hurricane Ida struck just as New Orleans was recovering from a peak in their COVID pandemic trajectory, with approximately 2500 patients hospitalized due to COVID and shortages of ICU beds. Against this backdrop, the jurisdiction, its Ryan White Program, HIV clinical providers, AIDS Service Organizations and community of PLWH were trying to respond to Ida. This participant, from a clinic that provided HIV and Hepatitis C prevention and care, described the toll of this “double whammy”:

After the hurricane, we were operating at about 50 percent capacity….We have 300 staff members and lots and lots of clinics, I don’t remember the exact statistics about how many people just quit because they didn’t have a home in Louisiana anymore, so they moved to other states or cities where they had family, but we did lose a lot of people...Going into the hurricane we lost [a staff member] on my team to COVID...And then, the medical assistant in the clinic was let go. So we were down to no medical assistants, and when that happens, the clinic is effectively closed because we can’t do labs or anything, we do fully telehealth and the clinic is closed. So going into Ida, the person who had known pretty much every single client by name…we didn’t have that person anymore. And, then the storm hits, we’re already down two people, and then another person on my team, she lived in [a hard hit area] and her home was destroyed. So we didn’t have her for, like, three weeks after... And, so, it was me and the case manager and [the doctor], but [the doctor] was working in the hospitals right after the storm [due to COVID]. So anyway, it was me and the case manager, we had to call every single patient and figure out where they were and what their medication needs were because lots of people just left without their meds, or they had a week left in their meds, or three days or something. And then we didn’t – normally at an organization [like our clinic], when you’re down a medical assistant, you can float from another clinic, but we were at 50 percent capacity, so we can’t float anybody. And then whenever we’re building back from Ida, there’s a new COVID surge, and then there’s another COVID surge. So there’s no floats anywhere, our clinic was closed for months. We had to send everybody to labs at our different clinics and then do telehealth appointments with them for their HIV care. And we just opened the clinic back up a month ago [5 months after the hurricane], and then our nurse – she got COVID and has been out for a month. It’s been a mess. [KI 02]

The organization was already strained due to COVID and then even though the agency did not experience substantial physical damage to the building, they did not have the staff capacity to keep their doors open following Ida. Months later, they were still struggling to re-engage with patients and resume in person clinic-based services.

Another clinic echoed this challenge, adding that they were in the process of adjusting to a new Electronic Medical Record (EMR) system on top of responding to COVID, and later the hurricane. The sense of constantly responding to waves of COVID followed by the impact of Ida added a sense of chaos as staff felt barraged by crisis after crisis.

I started [working here] in March of 2019. In November of 2019, we decided to migrate to a brand new EMR. So, we went from build-out to implementation in six weeks. Then the Christmas holidays….Then, of course, January, February, COVID hit, so then we had to figure out how to add the telehealth component to it. Then start adding all the other components with COVID on top of it. We have been juggling….And then, you know, Hurricane Ida was bad but we had several storms before that which would knock out our power, so it’s been kind of a constant step in motion to try and stay ahead of things. It’s one step forward, two steps back sometimes…Especially with COVID because, and this was across all of civilization, just about the time we were kind of thinking we had it under control, it came back again. And then, you know, Omicron and Delta [COVID variants] hit, and then Ida hit. Then we started over again. So, it’s been chaotic, it really has. [KI 06]

Similarly, another clinic noted that they just starting to get back to capacity after adjusting to COVID, when the “stupid storm hit.” Here, the informant alludes to the fact that they were without power at their home for over a week, and the clinic, even though it had a generator, was not able to maintain a steady power supply. Consequently, the clinic “lost everything” including vaccines and medications. After three weeks of closure, they were able to re-open but that patient population itself was totally displaced.

We were doing [retention] better. It was going up and up and up, and the stupid hurricane happened. You know, it’s really frustrating. I mean, it really screwed us. Because we lost, I had no power at my house for a week, a little over a week…At our larger clinic site, we had no power for even longer, and there was no power to this area of the city, where I am now, for a while. Our mid-city site got power back first, but our larger site also, the generator we had here is a diesel…and it would overheat, and then it would turn off to cool, then it would start back up. So, we lost all of our vaccines, all of our meds. It was just a mess. We had to close for two weeks and then the third week, open up slowly. And then, all these patients are everywhere and it was a mess...Because it just takes months to recover from that. And I feel like now, we’re starting to get back, and then omicron hit, but I think we’re back into a groove of at least seeing people as we normally were before Ida. [KI 07]

The challenges of navigating COVID along with the aftermath of Hurricane Ida made the recovery process especially long for clinics and agencies.

### Staff turnover, retiring and capacity issues post-Ida

The immediate and complicated impact of Ida on an already stressed workforce created a lot of psychological stress, which was felt within clinics but also reflected in the larger population of people living in the city, which struggled to resume normal services after the storm. This informant speaks about the lingering trauma, that makes it harder for people to “show up for work.”

In New Orleans, once the electricity came back on, most people try to kind of get back to life. But, of course, the trash not getting picked up and there is still this sense of... I think the trauma is the thing. It’s just traumatic for people. The fact that [Ida] was on the anniversary of Katrina, just the psychological stress, people are just shut down. I think a lot of people aren’t showing up for work right now, and it’s not because they can’t. They’re just too burned out by a sense of doom. So, I think it’s more that than anything else. So, you know, people stayed away longer than they needed to stay away, which meant they weren’t really available to do work. And it’s just a little bit the culture here right now. [KI01]

Several informants discussed the fact that the storm destroyed homes of their fellow staff members, or severely impacted their families. These employees were not able to come to work, leading the clinics to be short-staffed at a critical moment as they were trying to re-engage with patients that had scattered in the wake of evacuations. One key informant described the situation as “a mess.”

We pull the list [of patients who are out of care] every month. It’s usually around 100 people that are on, that show up on the list. We look at the list, we contact those people in a couple of different ways. Email, text, phone. Try to get them in and get them scheduled. If can get them, we schedule. If we can’t get them, we would submit it to the state for data to care matching. And then they would kind of, because so many people have shifted. I mean, New Orleans is like, it’s a mess. So, you know, especially after Ida, a lot of people were, like, done. Done. And so, moved to Florida, moved to Texas. [KI07]

With a number of patients permanently relocating to other states, it was difficult for clinics to reconcile their ongoing loss-to-follow-up lists. Exacerbating this situation, there were fewer employees to help track down the displaced patients. Like the patients, they also were displaced due to the impact of the storm.

We had some employees that lost everything, like, their homes were completely destroyed. Other employees were just kind of out for a few weeks until they got some water damage taken care of, so it was just a struggle to try to keep everybody up and running and functional, short-staffed. Our clients are the same way. We had a lot that were just completely upheaved but trying to make sure that they still received the care they needed and we had the medications, so it’s been a big project. [KI06]

Some of the staff members who left following the storm opted for early retirement and took the institutional memory of the clinics with them, also making it more difficult for the clinics to adequately train and onboard replacement staff given how quickly these employees had to leave.

[There is one case manager] that’s retiring. She has been with us almost 16 years… That’s like a big loss to lose her. But she is 62 and she’s retiring. She lost a lot in the hurricane. Her daughter had just got married and her daughter lost completely her house, like totally, won’t ever be able to go back. And the case manager, she had a lot of damage to her house. I think she was just like, “Let’s speed this up. I’m just going to go ahead and retire.” I don’t even think about stuff like that, but we’ve been impacted in ways like that as well…Lots of knowledge that she’s taking with her. [KI05]

Interestingly, in this case, the clinic hired a teacher who was actually burned out from weathering COVID and was looking for a change.

As the clinics were trying to locate patients who had been displaced, staffing shortages often thwarted their efforts, as health care workers and support staff were either unavailable to come to work due to coping with the aftermath of the storm, relocating or leaving the workforce. With the jurisdiction implementing the CHW model, some clinics found it helpful to have CHWs step in to try to track down displaced patients after hurricane, as the COVID pandemic continued.

Actually, not the hurricane necessarily, but the hurricane did provide us with some advantages – a lot of disadvantages, but some advantages… [Before] our community health workers were kind of stuck at the agencies to which they were assigned. Now they’re out there in the field as we had originally envisioned. They’re not just doing paperwork. They are out there meeting with clients, you know, and that’s – that’s opened a lot of doors and initiated dialogue that may not have occurred before…seeing a person face-to-face, meeting at a coffee house, that, you know, that says something. That says something to the individual. [KI03]

Still, some agencies were uncomfortable with the CHW model, and for them, CHWs became “just another set of hands” to help with retention. Some of the CHWs were people living with HIV and therefore there were some concerns regarding in person visits outside of the clinic during COVID, as this informant explains.

You know, [public health administrators] want people working nontraditional hours and hitting the bars and all that stuff, which you couldn’t do under the pandemic. A lot of it you could do, like make appointments to go to people’s houses and hang out on the porch with them…But a number of the community health workers [some of whom were living with HIV] were very uncomfortable with that from a health perspective and also uncomfortable with - you know, felt unsafe with that …. I think the original vision of people being out in the community did not really come to pass, and instead they are just working the phones. So, they’re basically linkage to care people. They’re really duplicating what a lot of agencies already have on staff. But since people are short-staffed, that’s helpful. But it’s not exactly a CHW program. [KI 01]

Still, the additional help from the CHW workforce was welcome in light of staffing limitations exacerbated by the hurricane.

### Physical damage and loss of physical infrastructure

Several of the clinics also suffered from physical damage to their buildings. In one case, the agency was in the process of renovating a new location and was able to maintain services and have their construction crew shift to take care of the more damaged building. Sadly, there were also instances where clinics had planned for hurricanes and extreme weather events in the aftermath of Katrina, yet still lost entire buildings and experienced displaced staff and services after Ida.

We thought we had a really good emergency plan. After Katrina, our CEO actually put a shower and a washer and dryer at the agency for us because so many people lived there after Katrina. Our CFO lived there for a few months after Katrina. So he put in a shower, washer and dryer, we already had two kitchens there. He got a full building generator for our main buildings. We coordinated off-site storage so all of our files after a certain time are stored off-site. We have all these mechanisms in place. But then when Ida came through, your generator doesn’t work when it floods. And your roof is blown off. It was upsetting. I mean, for me, I think, emotionally, it was really upsetting to see a place where that was like my home, you know? It was rough. It was really hard. [KI 05]

Despite the challenges of losing this building, the agency was able to continue services for their clients. Because they were a small agency, with a staff that had been working at the clinic for many years, the clients were able to stay connected to them despite the challenge of losing their building. The case management team in particular proved to be an indispensable part of their disaster response effort.

I feel like, for clients, it was able to run pretty seamlessly because most of our visits are home-based or community-based or at our food bank, and our food bank was fine…We did not lose touch with anyone. And I think that’s a benefit of us being small and all of us being here so long. My newest case manager has been with us for five years, which is still a pretty long time. [Our clients] reach out to us, you know, they’re like, “I’m in Texas. I’m coming back in three days.” They’ll tell us where they are. Most of our clients went to Texas or Mississippi if they had to leave. [KI 05]

For some, the ability to provide services via home visits or telehealth allowed their patients or clients to continue to experience continual care in the wake of Ida and its destruction.

### Maintaining access to treatment during Hurricane Ida

Probably the most pressing issue that many of our key informants faced related to HIV care was trying to help their patients, many of whom had been displaced, renew medications. This was especially challenging for those who were evacuated to settings across state lines. Fortunately, many informants were connected to one another via the QI collaborative and were able to share resources and support. Here, one informant noted that during the storm Medicaid allowed their in-house pharmacy to ship across state lines, which is not typically allowed, to meet the needs of their patients. Because the agency has this ability during emergencies, they are also incorporating this into planning with their patients for future events, having them switch to their in-house pharmacy so that they can continue to provide treatment via shipping.

[We are] switching as many of our patients to the [our agency’s] pharmacy as we can. Because it’s much easier for us…even if our pharmacy is closed down, we can tell our patients it’s closed down, but I can get this medication to you in two days. Or if we’re in New Orleans, we as employees are covered by our insurance and we can drive medication to people instead of them waiting for us to open or patients can use the pharmacy in Biloxi, [Mississippi]. So if everybody’s at our pharmacy, we ship. We can ship to anybody, anywhere. And during a storm, we can ship across state lines. So switching people over to our pharmacy is a big thing. And then making sure that we have emergency plans for everybody. And [our CHW] will work directly with our case manager on that project, too. [KI 02]

Here another informant discussed shipping across state lines, and the importance of creative workarounds, many of which had been developed during the pandemic to maintain care for people living with HIV.

When they suspended Medicaid shipping rules, that helped us. Because we’re lucky in Louisiana. We’re a Medicaid expansion state, but a lot of our patients are Medicaid recipients and [normally] you couldn’t ship meds across state lines. But they suspended that temporarily after the storm. So, we were able to get meds shipped to people. We did a lot of stuff with our in-house pharmacy shipping Meds and having to add pharmacies to the EMR. We have all these pharmacies all over the place now. So, we were able to do a good bit. I don’t have a good number on how many because we just would do them as audio visits…Just a lot of really necessary but creative kind of workarounds. It’s the only benefit of, you know, what the pandemic has shifted in terms of telehealth for things like... I mean, certainly, I would rather not have a pandemic. But at least it gave us some freedom to pivot a little bit more quickly for hurricane response. And be able to call people and we can bill for the visit, still. It’s legal. Because at some point, you know, you can only take so many losses. [KI 07]

The ability to ship medications, the flexibility of having electronic systems, including an EMR, and being able to provide and bill for care using telehealth all were vital as clinics sought to maintain access to treatment for their patients.

### Advanced planning for hurricanes and storms

Some clinics actively planned to mitigate the impact of Ida on patient care, taking lessons from having worked through Katrina. For these settings, a lot of processes and planning were in place. Staff indicated that while it was challenging at the time to try to meet people’s needs, the level of preparation in place helped patients tremendously during an otherwise challenging experience. Here an informant talks about the advance hurricane planning they do with their patients, while still noting that, even with planning, people were “caught off guard.”

For hurricane season, what we do is we start talking to our patients, hurricane season starts in June, so probably in April. We have a little pamphlet that we give to them because we learned a lot from Katrina. When our patients were scattered everywhere, with no access to medication - it was the same situation. I mean, Katrina had gone through Florida, we all thought it was done, and then the next thing we know, Saturday, she’s a five and coming towards us. So, they all left with three days of clothes, you know, we weren’t expecting it to be much like it was when Katrina hit. So, we learned from that you have to have a hurricane plan. We try to help our patients remember to get their medications, a nine-month supply, and we have like a little checklist for them to do…. We try to get everybody as prepared as you can be. And then everybody’s human nature. You know, Katrina was 16 years ago, and we really haven’t had anything as intense as that. You’re like, “Yeah, it’s another hurricane season...” but then Ida happens, and patients are caught off guard, we’re caught off guard, not hospital-wise but personal-wise. Because we always have the activation plan. We learned all that, so we try to get our patients to be that prepared too because it could happen and it did. [KI 04]

This informant then goes on to describe how the clinic and staff responded during Ida, with these plans in place. Even with the lessons of Katrina, there were still complications, because as they note, “whatever patients experience, we do too.”

And then Ida happened. A lot of people didn’t evacuate because, the Mayor, it was too close to call. It wasn’t a mandatory evacuation because of the way it happened. So, a lot of my patients wound up sheltering in place and that brings its own set of challenges because a lot of us were without power for 9, 10, 12 days. Ida hit on Sunday, the Katrina anniversary, the 29^th^. Then communication with all the cell phone towers and stuff was down. So, like it was spotty at best on Monday, getting better over the course of the week, but it depended on what carrier you have. Patients could have evacuated; they could have stayed; they were like us. It was like the staff. That’s the thing about living here too, is like, whatever our patients experience during these things, we do too. We have that connectivity that makes it, you know, rebuilding or experiencing it all. We share information with each other. The city was really good about, we have Recreational Development, they opened up for people to go charge their phones, get air conditioning because it’s still really hot at the end of August and September in New Orleans. So, for our patients, it could be any of those situations. And I learned this from Katrina, if you don’t have electricity, you can’t pump gas. If the gas stations weren’t on, you couldn’t get gas but then there were people panicking and getting gas and the grocery stores were closed. It was a different bad from Katrina. Katrina was all the floodwaters that we had for so long and the mold and the water staying in people’s homes forever, and this was wind and rain. No electricity. Getting food was hard for people. We were closed for two weeks but our providers were trying to call in because patients were trying to call in. Because we have a plan, a Code Gray, and we have activation teams that come on, but we’re the outpatient part of the world. So, clinics are closed but we can’t be closed for our patients. There’s a refill line that the patients can call to make sure they have their medications. If patients were calling in, people were putting messages to the doctors in their charts, our doctors were checking their patient messages from wherever they were, you know, if they stayed or if they decided to evacuate. [KI 04]

This clinic was able to continue to arrange for medications for their patients, partly because they had a refill line and so were able to maintain communications with their patients about these needs. In addition, providers had planned with many of their patients, so some had enough supply when they were evacuated. Other organizations relied on their relationships with other agencies and clinics to help sustain services for their clients.

In another case, an agency had a memorandum of understanding (MOU) in place with a “sister agency” which was outside of the path of the storm. That agency agreed to take their clients, and house them during the storm. Because of this plan, for their clients “it was business as usual.” There was roof damage to their [residential program’s] house in New Orleans and no power. And the residents could not stay in the house while the roof was repaired. Emergency planning and local collaborators made the arrangement work and minimized Ida’s impact on their clients, as this key informant describes.

Hurricane Ida. That was a bad one. As a whole, being that we work with the homeless population, they had no evacuation plan. …we have a memorandum of understanding with [another city], Louisiana, which is miles outside of – maybe a two-hour drive outside of New Orleans, and there’s a similar program that does what we do here in New Orleans, but in Lafayette. Due to the connection that our director has made, we were able to transport our clients to live in Lafayette for about a week or so. So, that’s what happened. They really had no place to go. [KI 08]

In another instance, a hospital-based clinic was able to make its pharmacy available to patients with RWHAP coverage from other clinics in the QI collaborative. RWHAP recipients were therefore able to go to the in-house pharmacy and get their HIV medications. Several other clinics, some of them involved in the collaborative, referred patients to this setting so they could maintain access to ART.

One of the clinics [in the QIC] had significant damage. The newer [hospital-based clinic in the QIC] wasn’t as damaged…We had to shut down because of power. The smaller clinic was mainly telehealth. I think we all kind of did it a little different, but it is nice to talk to other people [in the QI collaborative] because people know where they can get meds. We were able to talk about which pharmacies were open, what services were open, things like that. So, you know, a lot of the Ryan White recipients go to our in-house pharmacy... They were open until 7:00 or 8:00, every day of the week… a lot of people referred their patients over to get their meds, which was nice. [KI 07]

The relationships that the organizations had established through the QIC helped them navigate the intensity and aftermath of Hurricane Ida, through a variety of ways – from being able to evacuate their residents to sister agencies to being able to source medications at nearby pharmacies that were located in hospitals that still had power.

### The role of the QI collaborative: Desire for formally sharing resources following Hurricane Ida

During our interviews and observations at learning sessions in the wake of the hurricane, many informants expressed gratitude for having the collaborative to help support them and their clients during the hurricane. On the ground, the formal work of the collaborative shifted after Ida. As part of the natural progression of the QIC and jurisdictional capacity-building, the agency and clinic-based participants were given more ownership around the content for learning sessions, and were tasked with developing the agendas and planning for them. Accordingly, emergency planning became a central topic of these sessions.

Changing things up brought new perspectives and new focus. I think you need that sometimes…Because we’re trying to transition to, now, New Orleans [public health leadership] really just taking over QI work versus [the university] facilitating. One of the things that has happened with the learning sessions where we’ve done it every quarter, we’ve involved more people in the planning process. And that’s helped out a lot. I think it’s made people participate more. It’s made the learning sessions better. It’s more engaging. The challenge is to continue that on. [KI 09]

Another informant explained how the Collaborative ended up incorporating hurricane preparedness into its mission and thinking about lessons learned in the wake of Ida.

I think the push right now – the push across the entire city is hurricane preparedness and just, emergency preparedness. I think that that’s where [the collaborative is] going to go because I really think that a lot of health care providers and a lot of people in our field were kind of embarrassed by how this played out because we should have been more prepared. Like, this is New Orleans, it’s always going to happen again. I don’t think anybody has a reason to be embarrassed, but I think something was different about this storm maybe that people looked around and were, like, why did – why was this so hard? This shouldn’t have been so hard because we live in New Orleans. It’s not like a hurricane hit Oklahoma or something. Like, it’s Louisiana. We should know how to do this. And just that there weren’t necessarily, like, policies in place regionally and statewide for us to be able to assist our patients who had evacuated. So I think that that’s the way that [the collaborative] is going, and I think because there’s still so much recovery that has to happen, like, we’re still faced with it every day. I mean, we’re faced with it not just when you go to work. Like, obviously we see it when we go to work, and we still have clients that, you know, are in Florida and are waiting to come back to see us, but, I mean, you see it when you drive around the City of New Orleans; like, the stop light on my street turned back on last week. It’s January – it’s almost February and we just got our stoplight back. And [a colleague at another agency] and I were talking in the last learning session about how her trash hasn’t been picked up in, like, six months. So, people are still – like, you see it in the city and I think there’s just a push as a city as a whole to be, like, why were we so unprepared for this? And that’s spreading kind of across jurisdictions and, you know, everybody’s talking about it, I guess. [KI 02]

This informant goes on to talk about specific plans to establish and maintain communications so that patients in the NOLA jurisdiction receive better care during hurricanes and other emergencies. They stressed using existing programmatic infrastructure and relationships, sharing ideas and problem-solving during crises to better serve clients and weather future storms. In this way, the QIC supported both establishing new and strengthening existing relationships, resulting in better communication—which could be harnessed in future emergencies.

We brought up being able to talk across organizations [that are part of the QIC] as well, like having not only a plan for your patients but for staff. Like when something hits, we’ve already established maybe a Slack [online communication] channel of all of the leaders of the HIV organizations so that we can talk amongst each other and say, you know, my patient went to Walgreens in Biloxi, and the pharmacists denied services. Have you seen that, where else can they go, that kind of thing, and talk across organizations because we had no idea if we were all alone in this. Like, we had no idea if maybe our patients were just worse off than everybody else’s. Like, were they – were other people having this hard of a time? Were they contacting their patients like we were? We just had no idea what anybody else was doing…. it’s the people who are expected to do the work are also experiencing the disaster, so I don’t know how to get around that. Like having contacts, or a MOU is already set up with organizations maybe in North Louisiana or something that can do the work for you if you were also displaced, like, we send you a list beforehand and you maybe help us call our list or something. [KI 02]

Still, maintaining involvement in the QIC was stressful for some, especially those from smaller agencies, as they were contending with the complexities of COVID and recovering from Ida. As this informant notes, the Collaborative’s work took more of a back seat to responding to the more pressing issues.

I need to get [our agency’s involvement in the collaborative] back on track. It’s kind of, after Ida, I was like, “I don’t want to do this” because you get so behind. But that’s on the agenda for this week and next week, is to really get us reoriented, restructured. I think that’s the problem that I’ve discussed with our team, is you know, you start going on a project and it gets derailed. And then you start again, it gets derailed. And then, how many more times can you motivate your team to restart it?...Just like 1,000 questions. And you know, a nurse is out [with COVID] and tests positive still. You get distracted by those things. But I think the goal, simply, to answer your question, we need to restart the [QI] project [to re-engage patients who are out of care] again. [KI 07]

Despite the additional work of participating in the QIC, providers realized that it is value-added to facilitate achievement of the ultimate goal of re-engaging people who are out of care. This becomes especially relevant during and following extreme weather events and disasters.

## Discussion

Flooding, property destruction and loss of power following Hurricane Ida produced major disruptions in HIV care, including communication with healthcare systems, access to HIV medications and staff shortage. People were often forced to evacuate to other parts of the state or other states, and some were unable to relocate because of insufficient planning or resources. Specific difficulties centered around helping clients gain access to ART, especially at pharmacies across state lines in cases where individuals were evacuated. Because of staff shortages community health workers were asked to step in to help with finding clients and keeping them engaged in care. Participation in the QIC, using existing listservs and messaging apps allowed HIV-care teams to more effectively reconnect with clients, facilitate access to treatment, and feel less isolated and anxious as they tried to navigate the consequences of COVID while navigating the disruptions from the hurricane. Following the storm, the Collaborative came together to share emergency preparedness plans, including patient-oriented materials tailored for those living with HIV, as well as other lessons to ensure that patients who had been evacuated remained engaged in care and on treatment. Importantly, the design of the QIC, by including jurisdictional leadership, provided opportunities for direct interactions between program leaders and policy makers with providers and community members.

The impact of Hurricane Ida extended beyond the physical damage to buildings, power supply, city infrastructure and services, to seriously disrupt not only the lives of patients but also the lives of the people staffing clinics and community service organizations. As the US and other parts of the world contend with cyclical climate-related events, there is a need to consider the toll of these events on the healthcare workforce and devise strategies to both prepare for them and respond to them. As one informant put it, “*what impacts our patients impacts us.*” Trauma, as well as the material impact of health care workers losing homes, dealing with school closures, and navigating climate disasters themselves makes it imperative to proactively put mechanisms in place to better support the healthcare workforce during and in the aftermath of major climate events. Attention to provider and staff burnout, and retaining staff by providing remote work options and temporary housing if needed, childcare, flexible hours and workplace-based counseling and therapy may be important policies to consider to make the health care system more resilient in the face of climate change.

Although the CHW positions that were created as a result of the QIC were intended to be integrated into routine care, our study also shows how CHWs can be mobilized in health crisis situations [24]. CHWs are frequently called upon in this way. During the COVID-19 pandemic, there was a resurgence in funding for CHW positions to address health misinformation and promote vaccination and other preventive measures [25]. Unfortunately, many of those positions added during the height of the COVID-19 pandemic have now been cut [26], underscoring the precarity of these positions. However, NOLA was well positioned to respond quickly to the dual burden of the hurricane and COVID-19 due to their investment in CHW workforce development [27]. Having an existing and well supported CHW workforce who can respond and adapt is crucial for many reasons moving forward.

For those involved in the collaborative during and following Ida, sharing resources helped mitigate the immediate impact of the storm on the well-being of their patients. In some cases, clinics and agencies actively planned for hurricane season, establishing relationships and MOUs. This allowed them to evacuate clients out of harm’s way, ensure that patients had an adequate supply of medications following the storm, and communicate with other pharmacies and hospitals about medications and availability during and immediately following the storm.

Policy changes, such as the temporary suspension of rules that prohibit shipping of medications across state lines also proved to be vitally important as patients were dispersed across the region. In the future, these suspensions could be automatically triggered for regions during climate-related events, and continued so that clinics can respond quickly to support patients displaced across state lines whenever needed. Additionally, developing hurricane preparation plans, both for staff and for patients, including pointers such as what to include in a “go-bag” should patients face evacuation, also can help mitigate the impact of climate-related disasters.

Our participants demonstrated that there was a desire to further leverage the mechanism of a QIC to build on its structure and processes to take up disaster management and emergency preparedness more formally, recognizing that climate-related events occur on a regular, cyclical basis in NOLA. By definition, however, QICs are time-bound and address a specific topic [19]. Moving forward, the QIC could evolve into a Community of Practice or an ongoing QI Learning Network that would retain the types of communication channels that were so crucial in the HIV care system’s response to the aftermath of the hurricane, with periodic variation in intensity based on events. This case highlighted a novel way that a QIC was able to pivot and address an urgent, emerging issue while also connecting those new efforts to the initial priorities around improving HIV care services. Researchers and practitioners working in other areas of the health sector may benefit from adopting a similar approach to advancing other care outcomes while also responding to the threats from climate change. Strategies that formalize relationships, such as those established through QICs, but also through agency-to-agency, clinic-to-clinic agreements, hold great promise in creating the ability for the health care sector to be more responsive during the immediate crisis and in its aftermath. While comprehensive responses and active participation may be burdensome for smaller agencies that do not have the capacity to be at the table for lots of meetings, finding flexible, efficient ways to build networks and sustain them at the local level may contribute to better health outcomes in disaster prone areas. It may be possible to leverage resources to support QICs that may be available through additional funding related to climate resiliency, and to also involve local public health leadership to make these efforts more sustainable.

## Conclusions

The HIV health care system is particularly vulnerable to the impact of climate change. Beyond creating workforce resiliency and physical infrastructure, the need to develop emergency preparedness plans, particularly tailored for those living with HIV, and providing continuous access to treatment are crucial steps that require attention. QI Collaboratives create an effective vehicle for policymakers, health care providers and staff to share resources, coordinate services and strengthen communication during weather-related events, and should be considered in emergency preparedness planning efforts.
